# Characterizing Aircraft Exhaust Emissions and Impact Factors at Tianjin Binhai International Airport via Open-Path Fourier-Transform Infrared Spectrometer

**DOI:** 10.3390/toxics12110782

**Published:** 2024-10-28

**Authors:** Jingbo Zhao, Zixiang Mao, Bo Han, Zhiyong Fan, Simeng Ma, Jingxin Li, Rui Wang, Jian Yu

**Affiliations:** 1 School of Transportation Science and Engineering, Civil Aviation University of China, Tianjin 300300, China; jbzhao@cauc.edu.cn (J.Z.);; 2 Research Centre for Environment and Sustainable Development of Civil Aviation of China, Civil Aviation University of China, Tianjin 300300, China; 3 Engineering Techniques Training Center, Civil Aviation University of China, Tianjin 300300, China; 4 Civil Aviation Management Institute of China, Beijing 100102, China

**Keywords:** OP-FTIR, airport emissions, emission index, meteorology, aircraft age, real-time monitoring

## Abstract

The growth of the civil aviation industry has raised concerns about the impact of airport emissions on human health and the environment. The aim of this study was to quantify the emissions of sulfur dioxide (SO_2_), nitrogen oxides (NO_X_), and carbon monoxide (CO) from in-service aircraft via open-path Fourier-transform infrared (OP-FTIR) spectroscopy at Tianjin Binhai International Airport. The results suggest that the CO and NO_X_ emission indices (EIs) for five common aircraft/engine combinations exhibited substantial discrepancies from those reported in the International Civil Aviation Organization (ICAO) databank. Notably, during the idling, approach, and take-off phases, the CO EIs exceeded the ICAO’s standard values by (11.04 ± 10.34)%, (56.37 ± 18.54)%, and roughly 2–5 times, respectively. By contrast, the NO_X_ EIs were below the standard values by (39.15 ± 5.80)%, (13.57 ± 3.67)%, and (21.22 ± 4.03)% in the same phases, respectively. The CO and NO_X_ EIs increased by 31–41% and decreased by 23–24%, respectively, as the ambient temperature decreased from −3 °C to −13 °C. This was attributed to lower temperatures reducing fuel evaporation, leading to inefficient combustion and increased CO emissions and lowering the combustion temperature and pressure, resulting in reduced NO_X_ emissions. The CO EIs had a positive correlation with humidity (adjusted R^2^: 0.715–0.837), while the NO_X_ EIs were negatively correlated with humidity (adjusted R^2^: 0.758–0.859). This study’s findings indicate that humidity is a crucial factor impacting aircraft exhaust emissions. Overall, this research will contribute to the development of scientifically informed emission standards and enhanced environmental management practices in the aviation sector.

## 1. Introduction

According to the International Air Transport Association, total global air passenger traffic (in terms of revenue passenger kilometers or RPKs) increased by 36.9% in 2023 compared to that in 2022, returning to 94.1% of the pre-pandemic (2019) level. This statistic indicates that the global civil aviation industry has basically recovered to the pre-epidemic level, and the development trend is expected to continue in the future. However, this expansion has fueled public concerns about the health and environmental effects caused by airport emissions, which include carbon monoxide (CO), sulfur dioxide (SO_2_), nitrogen oxides (NO_X_), carbon dioxide (CO_2_), and ultrafine particulate matter [1,2,3,4]. Specifically, CO reacts with hydroxyl radicals, reducing their availability to participate in methane decomposition, which has an indirect effect on the greenhouse effect [5,6]. NO_X_ and SO_2_ could interact with atmospheric water vapor and oxygen to form nitric and sulfuric acids, respectively, which are components of what is commonly known as acid rain [7,8]. Additionally, prolonged exposure to high SO_2_ and NO_X_ concentrations is linked to increased incidences of respiratory and cardiovascular diseases, among other health complications [9,10,11,12]. Unfortunately, the continuous development of the civil aviation industry will inevitably cause increased emissions of various pollutants.

Given that such emissions contribute to the deterioration of air quality in the airport community, researchers have focused on quantifying aircraft exhaust emissions to assess the airport environmental quality of airports, provide insights into their sources, and propose emission reduction strategies [13,14,15,16,17,18]. Current airport emission inventories were mainly developed using fuel flow rates, operating hours, and emission indices (EIs) derived from the International Civil Aviation Organization (ICAO)’s emissions databank [19,20,21]. Therein, pollutant EIs are provided under different thrust settings of 7%, 30%, 85%, and 100%, which correspond to the taxiing, approach, cruising, and take-off phases, respectively [22]. However, interpreting these data as reflective of the actual emission values of aircraft is inaccurate [23,24,25]. Heland et al. (1998) utilized a mobile passive Fourier infrared spectrometer to monitor gaseous pollutant emissions of in-service passenger aircraft, discovering that the CO EIs for the CFM56-3 engine during taxiing were 27–48% higher than the ICAO data [26]. Klapmeyer et al. (2012) employed mobile vehicles to quantify NO_X_ EIs during taxiing, reporting that EIs were, at most, 25% lower than ICAO values [27]. Schäfer et al. (2003) revealed that the CO EIs for the A320 family of aircraft during the taxiing phase were approximately 38% higher than the ICAO standard value, while the NO_X_ EIs were ~78% lower [28].

This discrepancy is mainly attributed to ICAO standard values being provided by aircraft engine manufacturers who conduct bench tests on a limited number of new production engines and the internal and external factors that affect engines during such tests not accurately reflecting the actual operational environment [29,30,31]. Heland et al. (1998) posited that these discrepancies might have been strongly influenced by the maintenance and aircraft age [26]. Carslaw et al. (2008) studied the discrepancy in NO_X_ emission rates between the same engine types at London’s Heathrow Airport, reporting that NO_X_ emissions significantly varied by up to 28%, which was attributed to aircraft operational parameters such as the take-off weight and engine thrust settings [32]. By collecting the age information of target aircraft, Duan et al. (2022) revealed a weak positive correlation between NO_2_ and SO_2_ emissions and the age of Boeing 737–800 aircraft [33]. Furthermore, Zaporozhets et al. (2017) studied the effects of the fuel flow rate, engine service time, and ambient temperature on aircraft engine emissions of aircraft, reporting that the NO_X_ and CO emission concentrations of the A340-300/CFM56-5C2/F aircraft/engine combination were weakly correlated with the engine service time and ambient temperature and significantly and positively correlated with the fuel flow rate [34].

Various methodologies have been employed in previous studies to measure aircraft exhaust emissions, including the use of non-invasive instruments and mobile laboratories downwind of active runways. Heland et al. (1998) utilized a mobile passive Fourier-transform infrared (FTIR) measurement system located 20 to 40 m perpendicular to the exhaust stream, aiming to measure emissions from in-service aircraft [26]. Klapmeyer et al. (2012) arranged a mobile eddy covariance laboratory downwind of the aircraft runway and conducted field measurements only when the wind direction was suitable for capturing aircraft exhaust emissions during idling and take-off [27]. Considering the applicability of optical instruments, Schäfer et al. (2003) applied passive FTIR emission spectrometry to measure NO emissions, differential optical absorption spectroscopy to capture NO_2_ emissions, and active FTIR absorption spectrometry to detect CO emissions behind the nozzle [28]. Generally speaking, optical remote sensing monitoring methods demonstrate less background interference and exhibit good stability under varying environmental conditions, such as changes in wind direction and speed. Furthermore, such methods enable the collection of large samples while ensuring regular airport operation.

Consequently, a comprehensive experiment was carried out at Tianjin Binhai International Airport (TSN) from 29 November 2023 to 13 April 2024. During this period, the NO_2_, SO_2_, CO, and CO_2_ emissions from aircraft during the taxiing, approach, and take-off phases and their variations were captured via open-path Fourier-transform infrared (OP-FTIR) spectroscopy. A typical monitoring interval was selected as 13–22 December 2023, during which the NO_2_, SO_2_, and CO EIs for five aircraft/engine combinations were calculated based on actual measurements, revealing quantifiable discrepancies with ICAO standard values. Meteorological information and aircraft age were collected during the measurement period for further analysis. The specific objectives of this study were to (1) assess the environmental quality at TSN, (2) investigate the EIs of aircraft exhaust and their impact factors, and (3) recognize actual emission values and discrepancies with ICAO data.

## 2. Measurements and Methods

### 2.1. Test Airport

TSN is situated in the Dongli district of Tianjin, China, which is classified as a 4E class civil international airport, supporting the operation of four-engine, long-range, wide-body airliners such as the Boeing 747 and Airbus A340. It is a typical international airport in North China. TSN spans approximately 364,000 m^2^, with two parallel runways separated by 2100 m. Both runways are equipped to handle the take-off and landing demands of various large commercial aircrafts, where the western runway (16R/34L) is primarily designated for aircraft take-offs, while the eastern runway (34L/16R) is predominantly reserved for approaches. The airport community is predominantly green and devoid of major pollution sources, thereby minimizing the impact on measurement accuracy. The usage of the western runway during the observation period is shown in Table S1.

### 2.2. Emission Measurement System

Any activity that interferes with normal aircraft operation is prohibited, which has primarily contributed to the lack of research on exhaust emissions at airports. To ensure safe and accurate measurement of aircraft exhaust emissions, OP-FTIR spectrometry, which is characterized by being non-intrusive, having a long range, a high sensitivity, and high resolution (1 s intervals), was applied in this experiment.

**Figure 1:**
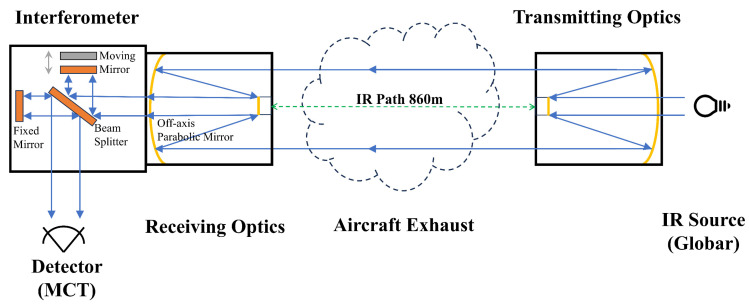
The schemes of the OP-FTIR system.

The spectrometer system comprised five components, as shown in Figure 1: an infrared (IR) source, the transmitting optics, the receiving optics, an interferometer, and a detector. The IR source emitted a wide spectrum of light, which was collimated into a parallel beam that entered the measurement area, allowing gas molecules to absorb specific wavelengths. Subsequently, this beam passed through the receiving optics into the interferometer, where a beam splitter divided it into two beams to generate interference. The detector converted the optical signals into electrical signals, which were processed by a computer using the FTIR algorithm to generate absorption spectrograms. OP-FTIR spectrometry was applied to the measurement of SO_2_, NO_2_, CO, and CO_2_ trace gasses.

**Figure 2:**
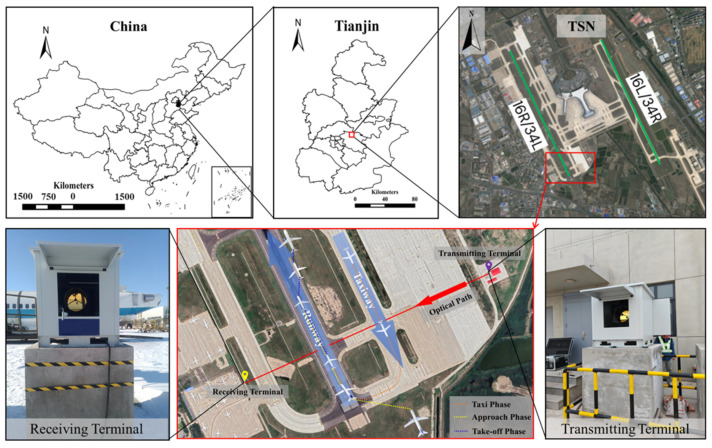
The location of TSN and the installation location of the OP-FTIR instrument.

The transmitting terminal, comprising the IR source and transmitting optics, was installed within the airport premises, and the receiving terminal, comprising the receiving optics, interferometer, and detector, was positioned at the apron of the Civil Aviation University of China with a total optical path length of 860 m (Figure 2). The spectrometer utilized a Globar IR light source, which is a robust source of IR radiation that is especially suitable for applications in the wavelength range of 100–6000 cm^−1^, to avoid disrupting the long optical path measurement. Moreover, the spectrometer utilized an off-axis parabolic mirror and mercury cadmium telluride (MCT) detector to enhance the signal quality by improving the signal-to-noise ratio and response speed. To ensure continuous and stable operation, the MCT detector was equipped with Stirling refrigeration technology, which effectively reduced the operating temperature, thereby improving its performance and sensitivity. Finally, 1.5 m high cement pillars were erected at both terminals to eliminate terrain-related influences on the optical path.

A recorder was installed at the receiving terminal to capture the taxiing, take-off, and approach times of aircraft. Meteorological data were obtained from the Meteorological Airport Database of the China Meteorological Administration, including wind speed, wind direction, temperature, dew point temperature, and sea pressure correction [35]. The wind rose map, which was derived from the wind speed and direction data at TSN from 0:00 on 13 December 2023 to 0:00 on 23 December 2023, is presented in Figure S1.

### 2.3. Data Processing Methods and Quality Control

Continuous monitoring at TSN was performed from 29 November 2023 to 13 April 2024. The OP-FTIR instrument was calibrated weekly using a specific standard gas to ensure its continued accuracy. Prior to calibration, pure nitrogen was introduced to purge the gas cell of the spectrometer and perform baseline calibration, minimizing background noise and the spectrometer’s own interference. Subsequently, the standard gas was diluted with pure nitrogen using a gas diluter, with dilution ratios of 10%, 30%, 40%, 50%, 60%, 70%, and 90%. These data were subjected to linear fitting against a theoretical model to evaluate the response characteristics and accuracy of the instrument, with the stipulation that a goodness of fit > 98% was required for the results to be deemed acceptable.

The exhaust emissions of aircraft passing through the optical path of the OP-FTIR instrument were captured during the monitoring period, as depicted in Figure S2. Due to parallel alignment of the taxiway and runway, simultaneous passage of take-off and taxiing aircraft was possible through the optical path. Data under such circumstances were excluded from the analysis. Additionally, the corresponding data were disregarded when ground service equipment at TSN, such as passenger cars, baggage and food carriers, container loaders, and cleaning vehicles, traversed the optical path.

**Figure 3:**
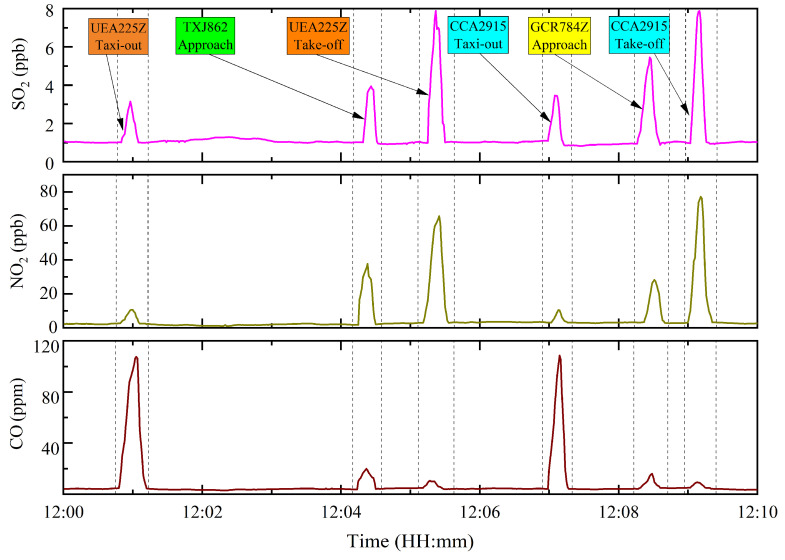
Real-time concentration curves of SO_2_, NO_2_, and CO measured from 12:00 to 12:10 on 16 December 2023 using OP-FTIR spectrometer.

Based on the abovementioned analysis, representative monitoring results were obtained from 13 December to 22 December in 2023. During this period, TSN experienced moderate aircraft traffic, minimal interference from ground service equipment, and favorable diffusion conditions, causing minimal disruption to measurement accuracy. Figure 3 illustrates the temporal variations in SO_2_, NO_2_, and CO emission concentrations in typical plume observations, which were representative of the trends observed throughout the entire monitoring period. The independent SO_2_, NO_2_, and CO peaks for almost every aircraft during the taxiing, take-off, and approach phases could be perfectly matched to the time at which the aircraft crossed the optical path of the OP-FTIR instrument. In the absence of aircraft in the optical path, the recorded data reflected the background environmental values at TSN. As aircraft passed through the optical path, the gas concentrations immediately increased to their maximum levels and then rapidly decreased to atmospheric background levels within a few minutes, representing the emission concentrations in aircraft exhaust after subtracting the background value.

Furthermore, the CO and NO_X_ EIs were derived from the concentration ratios measured within each aircraft plume during the taxi, approach, and take-off phases. It should be noted that since most NO in the exhaust plume and surrounding air was chemically transformed into NO_2_ immediately, the NO_2_ EIs detected were considered to be the NO_X_ EIs [27,28]. The EI could be calculated as follows:

(1) $$EI\left(X\right)=EI(CO_{2})\frac{c_{peak}\left(X\right)-c_{base}\left(X\right)}{c_{peak}\left(CO_{2}\right)-c_{base}\left(CO_{2}\right)}R(X)$$

where $EI(CO_{2})$ represents the emission index of CO_2_, calculated as 3150 g per kilogram of fuel, assuming a constant carbon content in jet fuel and complete conversion to CO_2_; $c_{peak}$ denotes the maximum concentration observed within the aircraft plume, presented in ppm or ppb; $c_{base}$ referres to the background concentration at the airport, presented ppm or ppb; and R(X) represents the molecular weight ratio of a given element *X* relative to CO_2_, presented as a dimensionless constant.

## 3. Result and Discussion

### 3.1. Airport Air Quality

**Figure 4:**
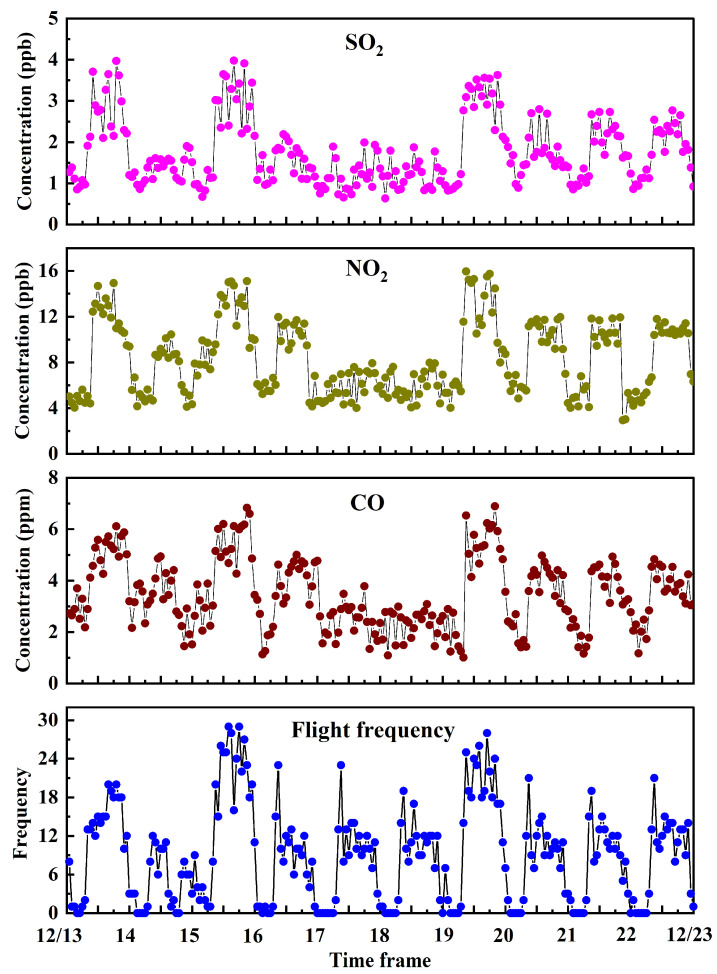
The one-hour mean concentrations of SO_2_, NO_2_, and CO in the measured optical path and the sorties of aircraft take-offs and landings over the target runway (13 December 2023 to 22 December 2023).

Figure 4 displays the hourly mean concentrations of SO_2_, NO_2_, and CO alongside the number of aircraft movements throughout the measurement period, with ranges of 0.63–3.98 ppb (median: 1.54 ppb), 2.94–15.96 ppb (median: 7.60 ppb), and 1.01–6.89 ppm (median: 3.24 ppm), respectively. The maximum hourly mean concentrations of SO_2_, NO_2_, and CO during the observation period were significantly lower than the Class 11 h mean concentration limits of current ambient air quality standards in China (150 µg/m^3^, 200 µg/m^3^, and 10 mg/m^3^, respectively). The low pollution levels observed were primarily attributed to favorable meteorological conditions that facilitated rapid dispersion of aircraft-emitted pollutants and the lack of other meaningful pollution sources in the airport vicinity.

This study’s findings are generally consistent with those of Duan et al. (2022), who utilized a long-range differential absorption spectrometer at Hefei Xinqiao International Airport, reporting hourly mean concentrations of SO_2_ and NO_2_ with ranges of 1.4–3.6 and 4.0–16.1 ppb, respectively [33]. Additionally, Schürmann et al. (2007) performed two in situ measurements and two open-path measurements at the Zurich airport, reporting median NO_2_ concentrations of approximately 10.6, 9.5, 12.2, and 16.0 ppb, respectively, which were slightly higher than the observed concentrations at TSN [36]. The measured CO concentration ranges in the taxiway and apron for the Zurich airport were ~0.05–3.92 and 0.16–1.67 ppm, which were lower than those observed at TSN, as presented in Table 1. This discrepancy was mainly due to the measurement results being affected by the aircraft activity levels, monitoring site location, and meteorological conditions.

As shown in Figure 4, the pollutant concentrations at TSN matched the number of aircraft flights. Valotto et al. (2016) employed a statistical model to characterize the hourly atmospheric concentrations of NO_X_ near Venice International Airport, reporting that aircraft exhaust emissions had a limited impact on the environmental NO_X_ concentrations despite aircraft being a statistically significant source, and road traffic may have been the main source near the sampling point [38]. Helmis et al. (2011) studied the meteorological effects on background air quality at Athens International Airport, pointing out that low background wind conditions resulted in the development of local flows over the greater area that preserved high pollutant concentrations. The strong background flow reduced the diurnal cycle of pollutant concentrations by >50% owing to advection and subsequent mixing of the lower atmosphere [37].

Therefore, the effects of the sampling site location, environmental conditions, aircraft activity levels, and background concentrations should be taken into account when monitoring and assessing for emissions.

### 3.2. Emission Index

Over the 10 days of sampling at TSN, the NO_X_ and CO emissions for different aircraft types during the taxiing, take-off, and approach phases were captured to calculate their EIs. A comprehensive understanding of the engine type equipped on each aircraft model is essential to compare the ICAO data with actual emissions. However, due to confidentiality practices among aircraft manufacturers, specific engine types installed on aircraft are generally undisclosed, and engine types installed on the same aircraft model may also differ. Herein, the A320-214/CFM56-5B4, B737-700/CFM56-7B26, B737-800/CFM56-7B27, A320-232/V2527-A5, and CRJ900/CF34-8C5 aircraft/engine combinations (the following is abbreviated as aircraft model) were selected to represent the most commonly used and widely adopted models in the market.

The measured gas pollutant EIs across various aircraft/engine combinations and operational phases are listed in Table 2. The data indicate that the NO_X_ EIs increased by 5–15 times as thrust increased (aircraft pushing from taxi to take-off), while the CO EIs decreased by 15–30 times. For instance, the NO_X_ EIs for A320-214 during the taxiing phase were 3.06 ± 1.66 g/kg of fuel, which escalated to 8.14 ± 1.60 g/kg of fuel during the approach phase, and further surged to 22.77 ± 3.67 g/kg of fuel during the take-off phase. This result was attributed to NO_X_ production being derived not only from fuel combustion but also from nitrogen combustion in the combustion chamber. The temperature and pressure within the combustion chamber increase under heightened aircraft thrust conditions, promoting the conversion of N_2_ to NO_X_ [39,40]. As a low-thrust aircraft/engine, CRJ900 exhibited significantly lower NO_X_ EIs during take-off than other short- to medium-range narrow-body aircraft (50%), while this trend was not significant at low thrust.

Conversely, the CO EIs for A320-214 significantly decreased from 33.23 ± 5.48 g/kg of fuel during taxiing to 3.66 ± 1.75 g/kg of fuel during approach and further decreased to 1.00 ± 0.72 g/kg of fuel during take-off. This trend suggests that combustion was incomplete under low thrust conditions primarily due to the relatively high fuel-to-oxygen ratio in the combustion chamber, which reduced the contact area between fuel and oxygen, preventing complete combustion and thus leading to increased CO formation [22,40]. The CO emission concentration of CRJ900 at low thrust was significantly lower than that of other aircraft; however, a similar trend was not observed at high thrust.

**Figure 5:**
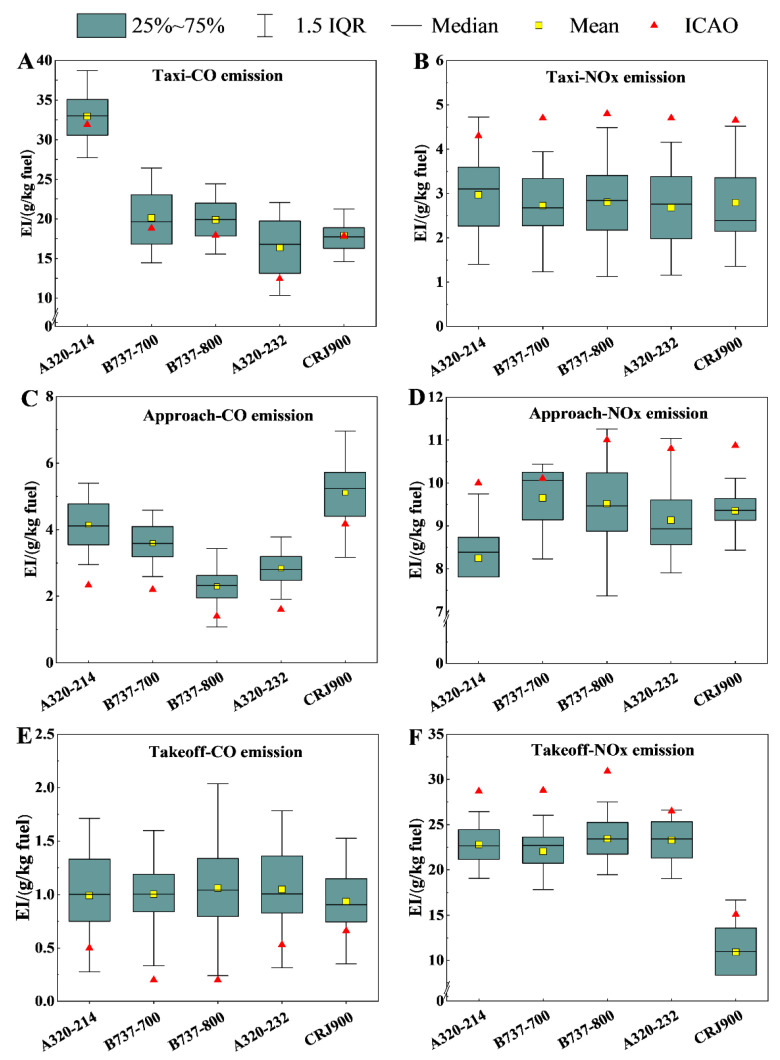
Comparison of EIs for CO and NO_X_ measured during taxiing, approach, and take-off phases with ICAO standard values; (**A**,**C**,**E**) correspond to CO EIs during taxiing, approach, and take-off phases; (**B**,**D**,**F**) correspond to the NO_X_ EIs during taxiing, approach, and take-off phases, respectively.

Figure 5 presents the box plots for the measured EIs and specific ICAO standard values. The measured mean CO EIs during the taxiing, approach, and take-off phases were generally higher than the ICAO’s standard values, while those for NO_X_ were lower than the ICAO’s standard values. The measured CO EIs exceeded the ICAO’s standard values by (11.04 ± 10.34)% during the taxiing phase, by (56.37 ± 18.54)% during the approach phase, and by (219.11 ± 173.54)% during the take-off phase. The NO_X_ EIs were (39.15 ± 5.80)%, (13.57 ± 3.67)%, and (21.22 ± 4.03)% below the ICAO’s standard values during the taxiing, approach, and take-off phases, respectively. These trends are consistent with the research findings of Haland et al. (1998) [26] and Kapmeyer et al. (2012) [27].

The discrepancy between the actual measurements and the ICAO’s standard values primarily stemmed from three key factors: the fuel type, the environmental conditions, and engine usage. First, the fuel types used in actual operational settings may differ from those utilized during bench testing for certification purposes, and the different physical properties of fuels influence fuel combustion and exhaust emissions. Kroyan et al. (2022) demonstrated that an increase in fuel density reduced fuel consumption, and a higher hydrogen content enhanced fuel consumption. Fuel viscosity did not have a significant impact on fuel consumption but ensured the proper atomization of fuel and evaporation of droplets at low temperatures, which maintained continuous combustion during engine operation [41]. Second, variations in environmental factors, such as temperature, humidity, and atmospheric pressure, affect the combustion process in the engine combustion chamber during operations, leading to emission variations [29]. Third, with the prolonged operation of the engine, the combustion efficiency of the engine declines with prolonged operation, resulting in the generation of more CO and limiting the conversion of N_2_ into NO_X_ [34]. Consequently, the measured CO and NO_X_ EIs deviated from the standard values in the ICAO databank. Precise measurements of in-service aircraft across various states, environments, and service durations are imperative for the acquisition of accurate emission data.

This study’s findings suggest that a variety of factors greatly influence the levels of gaseous pollutant emissions. Consequently, available real-time data, including ambient temperature, humidity, and aircraft age, were employed to investigate their impacts on aircraft exhaust emissions.

### 3.3. Impact Factors

The results presented above suggested that a variety of factors had a significant influence on the levels of gaseous pollutant emissions. Consequently, available real-time data, including ambient temperature, humidity, and aircraft age, were employed to investigate the impact on aircraft emissions in this research segment.

#### 3.3.1. Meteorological Conditions

The influence of meteorological conditions on aircraft exhaust emissions was investigated with the imperative of controlling relevant variables and ensuring a sufficient sample size. Each aircraft was identified by its aircraft registration number, and the aircraft models with the highest numbers of aircraft (i.e., sample size) were determined. Two types of aircraft were chosen based on the above selection criteria, with the registration numbers B5213 and B1219, which corresponded to aircraft models B737-700 and B737-800 and sample sizes of 9 and 14, respectively.

**Figure 6:**
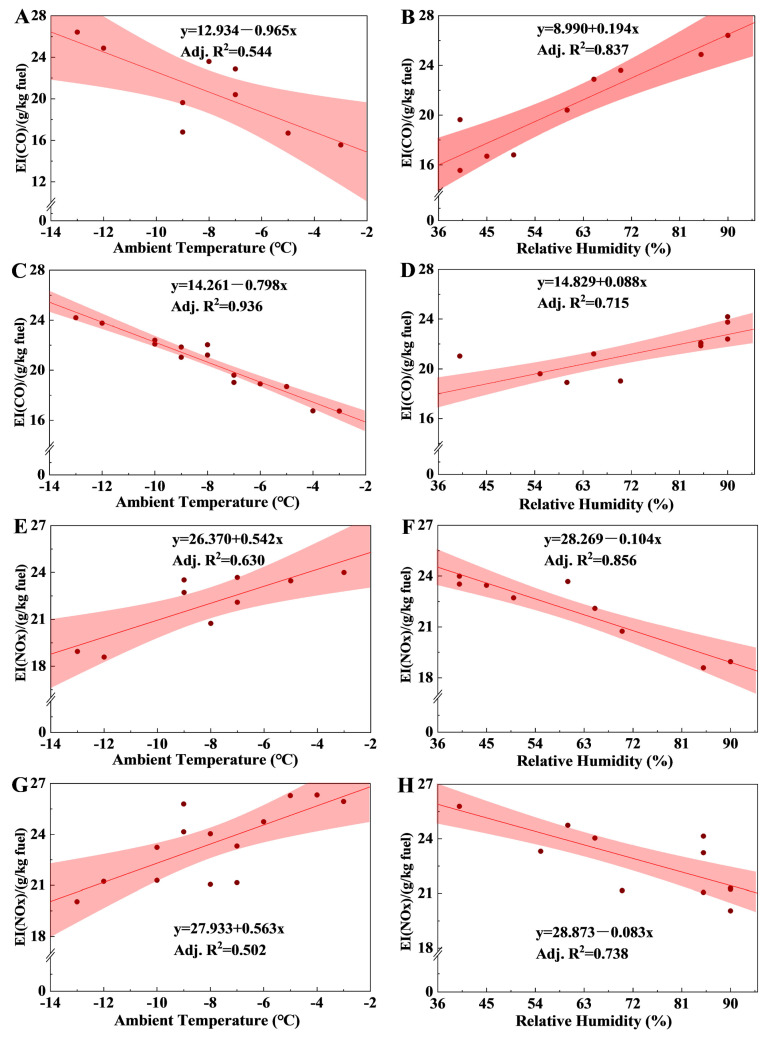
The correlation between exhaust EIs with ambient temperature and relative humidity during the taxiing phase; (**A**,**B**) correspond to the CO emitted from aircraft B5213 at the taxiing stage; (**C**,**D**) correspond to the CO emitted from aircraft B1219 at the taxiing stage; (**E**,**F**) correspond to the NO_X_ emitted from aircraft B5213 at the take-off phase; and (**G**,**H**) correspond to the NO_X_ emitted from aircraft B1219 at the take-off phase.

The variations in CO EIs during taxiing with air ambient temperature and relative humidity are illustrated in Figure 6. The correlations (adjusted coefficient, R^2^) between the CO EIs and ambient temperature were 0.544 and 0.936 for the B5213 and B1219 aircraft, respectively. The CO EIs increased by approximately 41% and 31% for the B5213 and B1219 aircraft, respectively, as the temperature decreased from −3 °C to −13 °C. This trend was consistent with the results of the study by Turgut et al. (2015), who performed gas emission measurements on 11 CFM56-7B26 turbofan engines and revealed that a higher ambient temperature contributed to lower CO emissions [29]. Similarly, Zaporozhets et al. (2017) concluded that CO EIs increased by approximately 30% when the temperature decreased from 20 °C to 10 °C [34]. Notably, the air density increases as the ambient temperature decreases, promoting engine intake and power output; however, cold air reduces the fuel evaporation rate, resulting in poor combustion and increased CO emissions [42]. Similarly, the CO EIs during taxiing were significantly and positively correlated with relative humidity for the B5213 and B1219 aircraft, with adjusted R^2^ values of 0.837 and 0.715, respectively. Hakan et al. (2023) revealed that humid air decreases the combustion temperature of fuel, reducing combustion completeness due to the specific heat of air, thereby changing its heat absorption [42]. In terms of road traffic, Hall et al. (2020) reported that CO emissions from petrol vehicles had low sensitivity to temperature, which was attributable to good emission control technology in petrol cars [43]. Meanwhile, Wang et al. (2015) found that an elevated intake temperature could increase the temperature in the cylinder of diesel engines, accelerating the CO–CO_2_ oxidation reaction and reducing the incomplete combustion of the mixture, thus effectively reducing CO emissions [44]. However, CO emissions for marine diesel engines were increased with increasing intake air temperature and humidity, which impacted the combustion process inside the engine and emission formation [45].

NO_X_ emissions were also substantially affected by ambient temperature and humidity. The NO_X_ EIs during the take-off phase were positively correlated with ambient temperature (adjusted R^2^ values of 0.630 and 0.502 for the B5213 and B1219 aircraft, respectively) and negatively correlated with humidity (adjusted R^2^ values of 0.859 and 0.738, respectively). The NO_X_ EIs were reduced by approximately 23% and 24% for the B5213 and 0.738 aircraft, respectfully, as the ambient temperature decreased from −3 °C to −13 °C. The same phenomenon was observed in the measurement of aircraft exhaust emissions at International Borsippier Airport, where the NO_X_ EIs were reduced by approximately 26% when the ambient temperature decreased from 30 °C to 20 °C [34]. This was attributed to the positive correlation between ambient temperature and engine performance parameters such as exhaust and combustion chamber inlet temperatures, which, in turn, affect the N_2_–NO_X_ oxidation reaction. However, in terms of road mobile sources, NO_X_ emissions from diesel trucks decreased by approximately 50% as the ambient temperature increased from 5 °C to 25 °C, while NO_X_ emissions from petrol vehicles exhibited no significant trend with ambient temperature [43]. Weilenmann et al. (2009) investigated the cold-start emissions of modern passenger cars at different low ambient temperatures, reporting that NO_X_ emissions of diesel vehicles exhibited a clear upward trend with a decreasing temperature, while no clear trend was observed in the emissions of gasoline vehicles [46]. These studies demonstrate distinct differences in the impact of air temperature on NO_X_ emissions across diesel and gasoline engines and aviation engines. Notably, NO_X_ emissions are also sensitive to humidity, as demonstrated by Aygun et al. (2023) [42]. Generated under high humidity, water vapor can interact with NO_X_, reducing NO_X_ production. Pirola et al. (2020) explored the effect of moist gas on NO_X_ emission levels under different oxygen concentrations, concluding that moist ambient air significantly reduced NO_X_ emissions under oxygen-rich conditions while maintaining thermal efficiency and soot reduction benefits. Under normal oxygen conditions, increased humidity resulted in a reduction in the indicated mean effective pressure in the cylinder, leading to decreased NO_X_ emissions [47]. Thus, the study findings suggest that airlines can optimize their flight schedules based on meteorological forecasts of ambient temperature and humidity to reduce CO and NO_X_ emissions. For emission models, emission data could be corrected by meteorological conditions to obtain accurate airport emissions.

#### 3.3.2. Aircraft Age Conditions

**Figure 7:**
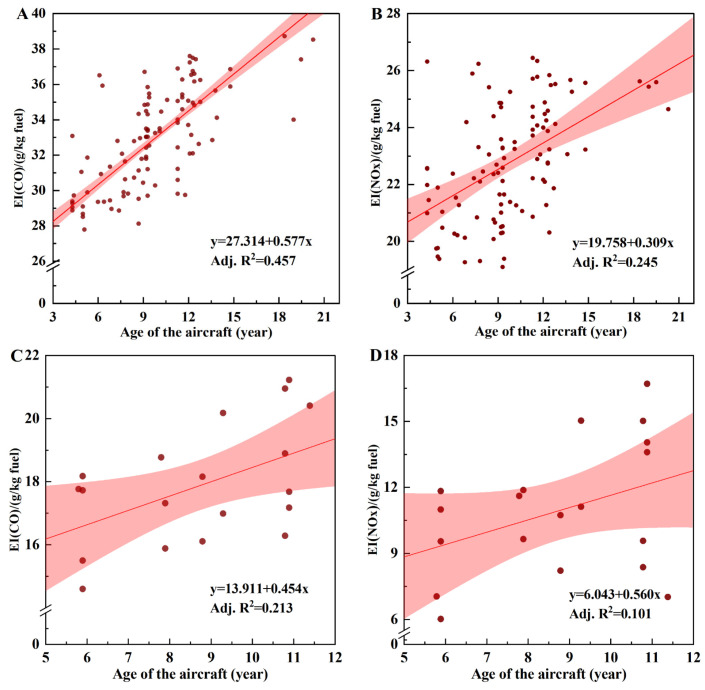
The correlation between exhaust EIs with the age of the aircraft: (**A**) the CO emitted from aircraft A320-214 at the taxiing stage; (**B**) the NO_X_ emitted from aircraft A320-214 at the take-off stage; (**C**) the CO emitted from aircraft CRJ900 at the taxiing stage; and (**D**) the NO_X_ emitted from aircraft CRJ900 at the take-off stage.

Figure 7 demonstrates the linear dependencies between aircraft age and CO EIs during the taxiing phase and NO_X_ EIs during the take-off phase, which were fitted at a 95% confidence interval. Weak dependencies were observed between the EIs and aircraft age, with R^2^ values of 0.457 for CO and 0.245 for NO_X_ for the A320-214 aircraft and R^2^ values of 0.213 for CO and 0.101 for NO_X_ for the CRJ900 aircraft. In the studies by Zaporozhets et al. [34] (2017) and Duan et al. [33] (2022), there were consistent correlations between aircraft age and CO and NO_X_ EIs. These weak correlations were attributed to several factors. First, they were attributed to the aircraft age obtained from the official reported duration of service, which does not exactly correspond to the usage time of an aircraft’s engines as airlines regularly perform maintenance and occasionally replace these engines [33]. Second, weather conditions vary during flights, further weakening the correlation between emissions and aircraft age.

This study’s findings revealed an increase in the CO and NO_X_ EIs with increasing aircraft age. This trend was attributed to a prolonged usage of engines, which accumulate wear despite regular maintenance, leading to decreased combustion efficiency and increased CO and NO_X_ emissions [2,48]. Sogut et al. (2017) reported that the degradation of aircraft turbofan engine performance directly affects entropy generation, thereby reducing the potential for engine improvement and leading to increased emissions [49]. Agarwa et al. (2011) noted that diesel engine aging deteriorated engine emission performance by increasing carbon deposits and piston ring wear, leading to increased emissions of particulate matter, hydrocarbons, CO, and NO_X_ [50]. Additionally, the accuracy of engine control system sensors diminishes over time, impacting the optimal operation of the engine and indirectly increasing emissions [2].

## 4. Conclusions

This study captured the changes in the SO_2_, NO_2_, and CO concentrations at TSN and analyzed pollutant EIs from aircraft emissions. The conclusions of this study are as follows:

(1) During the observation period, the average hourly SO_2_, NO_2,_ and CO concentrations were 0.63–3.98 ppb (median: 1.54 ppb), 2.94–15.96 ppb (median: 7.60 ppb), and 1.01–6.89 ppm (median: 3.24 ppm), respectively, which were much lower than the Class 11 h mean concentration limits of the current ambient air quality standards in China.

(2) The CO EIs decreased, and the NO_X_ EIs increased in conjunction with an increase in operational thrust. The CO and NO_X_ EIs measured from actual operating aircraft significantly differed from the ICAO’s standard values during different phases. During the taxiing phase, the measured CO EIs exceeded the ICAO’s standard values by (11.04 ± 10.34)%, increasing to (56.37 ± 18.54)% during the approach phase and (219.11 ± 173.54)% during the take-off phase. By contrast, the NO_X_ EIs were below the ICAO’s standard values by (39.15 ± 5.80)%, (13.57 ± 3.67)%, and (21.22 ± 4.03)% in the taxiing, approach, and take-off phases, respectively.

(3) The CO EIs increased by 31–41% during the taxiing phase as the air temperature decreased from −3 °C to −13 °C, whereas the NO_X_ EIs decreased by 23–24% during the take-off phase. Compared to aero-engines, the impact of temperature on NO_X_ emissions from diesel and gasoline engines was significantly disparate.

(4) The CO EIs were positively correlated with humidity (adjusted R^2^: 0.715–0.837), illustrating that humid air could affect the completeness of combustion. However, the NO_X_ EIs exhibited a reverse trend (adjusted R^2^: 0.758–0.859), which was mainly attributed to NO_X_ reacting with water vapor, thus reducing NO_X_ emissions.

(5) Aircraft age was weakly correlated with CO and NO_X_ EIs likely due to factors such as periodic maintenance and engine replacements conducted by airlines, which do not directly reflect the aircraft’s engine usage time.

Owing to the unavailability of certain information, we were unable to ascertain the engine types of the remaining aircraft models, which restricted comparisons to standard values. Similarly, we could not precisely estimate the impact of aircraft age on emissions due to confidentiality among aircraft manufacturers regarding the engine types installed on aircraft.

In future work, gathering more comprehensive data will enable a more complete study of aircraft exhaust emissions. Additionally, sustained long-term monitoring will provide valuable insights into variations in airport air quality over time.

## Figures and Tables

**Table 1:** Literature review of pollutant concentration by comparing previous major studies with this work.

Author	Instrument/Method	Sampling Point	Meteorological Condition	Pollutant Concentration			
				CO	NO	NO_2_	SO_2_
Duan et al. (2022) [33]	Long-path DOAS instrument	Hefei Xinqiao International Airport	-	-	-	4.0–16.1 ppb (Median: 8.2 ppb)	1.4–3.6 ppb (Median: 2.1 ppb)
Schürmann et al. (2007) [36]	Long-path FTIR and DOAS instruments	Pier A (designated for parking and simultaneous handling of up to four aircrafts)	-	-	Bdl*–61.1 µg/m^3^ (Median: 3.1 µg/m^3^)	3.6–103.8 µg/m^3^ (Median: 19.6 µg/m^3^)	-
		Pier B (designated for long-term parking of aircrafts with no handling taking place)	-	-	Bdl*–76.3 µg/m^3^ (Median: 6.6 µg/m^3^)	3.0–98.3 µg/m^3^ (Median: 17.5 µg/m^3^)	-
		Taxiway	-	0.06–0.49 mg/m^3^ (Median: 0.19 mg/m^3^)	0.07–132.0 µg/m^3^ (Median: 15.4 µg/m^3^)	0.04–274.0 µg/m^3^ (Median: 22.6 µg/m^3^)	-
		Handling (airport apron area mainly impacted by ground support vehicle emissions)	-	0.00–1.91 mg/m^3^ (Median: 0.22 mg/m^3^)	0.8–636.0 µg/m^3^ (Median: 25.2 µg/m^3^)	0.3–131.0 µg/m^3^ (Median: 29.6 µg/m^3^)	-
Helmis et al. (2011) [37]	Mobile monitoring stations	Athens International Airport	Moderate surface flow (wind speed: 7–9 m/s at 10 m height)	0.06–0.68 mg/m^3^ (Median: 0.18 mg/m^3^)	1.0–42.0 µg/m^3^ (Median: 92.0 µg/m^3^)	5.0–78.0 µg/m^3^ (Median: 21.0 µg/m^3^)	1.0–18.0 µg/m^3^ (Median: 10.0 µg/m^3^)
			Strong surface flows (wind speed: 9–15 m/s at 10 m height)	0.06–0.15 mg/m^3^ (Median: 0.09 mg/m^3^)	0.06–56.0 µg/m^3^ (Median: 22.1 µg/m^3^)	2.0–21.5 µg/m^3^ (Median: 5.0 µg/m^3^)	1.0–16.0 µg/m^3^ (Median: 8.5 µg/m^3^)
This study	Long-path FTIR instrument	Tianjin Binhai International Airport	Wind speed: 1–4 m/s; dominant direction: 330°	1.01–6.89 ppm (Median: 3.24 ppm)	-	2.94–15.96 ppb (Median: 7.60 ppb)	0.63–3.98 ppb (Median: 1.54 ppb)

* Bdl: below detection limit of 4 mg/m^3^ for NO.

**Table 2:** The NO_X_ and CO EIs for the 5 aircraft/engine combinations in different operating phases.

Combinations	Phase	EI (NO_X_) (g/kg Fuel)	EI (CO) (g/kg Fuel)	Sample Size
A320-214/CFM56-5B4	Taxi	3.06 ± 1.66	33.23 ± 5.48	104
	Approach	8.14 ± 1.60	3.66 ± 1.75	65
	Take-off	22.77 ± 3.67	1.00 ± 0.72	104
B737-700/CFM56-7B26	Taxi	2.59 ± 1.36	20.44 ± 5.97	46
	Approach	9.34 ± 1.11	3.59 ± 1.00	17
	Take-off	21.94 ± 4.11	0.97 ± 0.64	46
B737-800/CFM56-7B27	Taxi	2.81 ± 1.69	20.01 ± 4.44	567
	Approach	9.31 ± 1.95	2.26 ± 1.18	188
	Take-off	23.48 ± 4.02	1.14 ± 0.90	567
A320-232/V2527-A5	Taxi	2.66 ± 1.50	16.19 ± 5.86	74
	Approach	9.48 ± 1.57	2.85 ± 0.94	30
	Take-off	22.84 ± 3.77	1.05 ± 0.74	74
CRJ900/CF34-8C5	Taxi	2.94 ± 1.58	17.90 ± 3.32	19
	Approach	9.34 ± 0.82	5.09 ± 1.97	8
	Take-off	11.35 ± 5.37	0.94 ± 0.59	19

## Data Availability

The data presented in this study are available on request from the corresponding author.

## Supplementary Materials

The following supporting information can be downloaded at https://www.mdpi.com/article/10.3390/toxics12110782/s1, Table S1: The usage of the target runway during the observation period; Figure S1: Wind rose diagram of TSN from 0:00 on 13 December 2023 to 0:00 on 23 December 2023; Figure S2: Three scenarios of aircraft near the ground passing through the light path at the airport.
