# Supplementary material for: Characterizing Aircraft Exhaust Emissions and Impact Factors at Tianjin Binhai International Airport via Open-Path Fourier-Transform Infrared Spectrometer
Source: Toxics. 2024 Oct 28;12(11):782. doi: 10.3390/toxics12110782 (PMC11598071; doi:10.3390/toxics12110782)
Supplement: Supplementary file 1 [file toxics-12-00782-s001.zip › toxics-3249905-supplementary.pdf]

toxics

Supplementary materials

Characterizing Aircraft Exhaust Emissions and Impact Factors at Tianjin Binhai International Airport via Open-Path Fourier-Transform Infrared Spectrometer

Jingbo Zhao [1,2], Zixiang Mao [1,2], Bo Han [1,2,*], Zhiyong Fan [3], Simeng Ma [1,2], Jingxin Li [1,2], Rui Wang [1,2] and Jian Yu [2,4]

Number of pages: 4

Number of figures: 2

Number of tables: 1

Table S1. The usage of the target runway during the observation period.

Date	Usage of target runway
12.13	A, C
12.14	A
12.15	A, C
12.16	A
12.17	B
12.18	B
12.19	A, C
12.20	A
12.21	A
12.22	A

A indicates the aircraft take-off phase from the target runway in a south-to-north direction; B indicates north-to-south take-off; C refers to a south-to-north approach on the target runway.

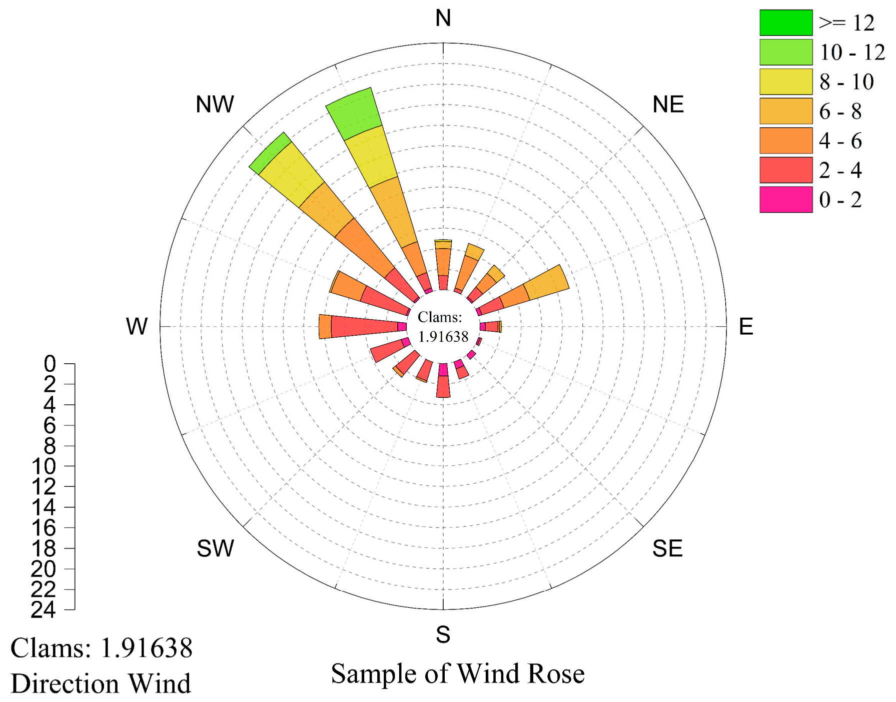

Figure S1. Wind rose diagram of TSN from 0:00 on 13 December 2023 to 0:00 on 23 December 2023.

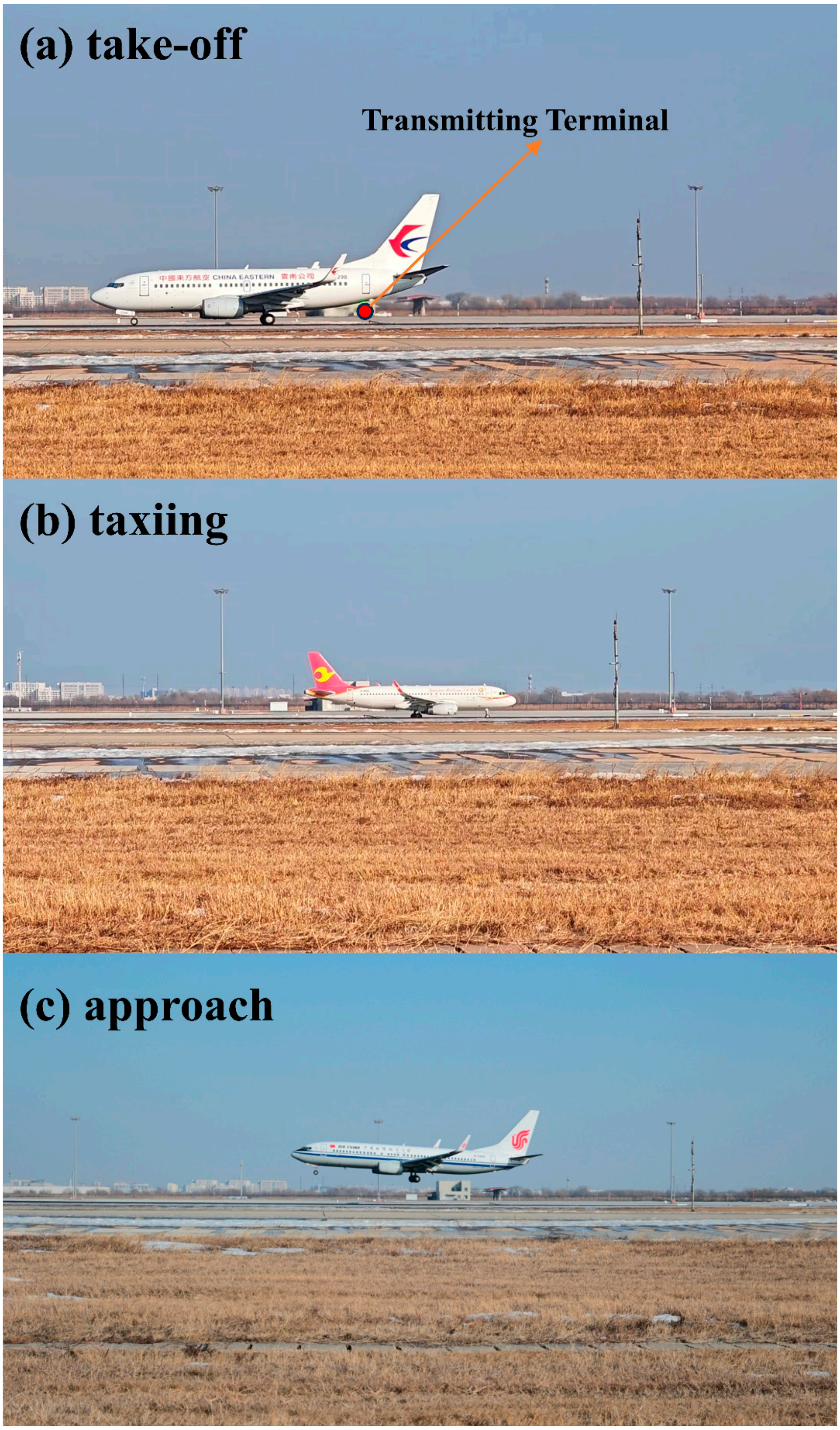

Figure S2. Three scenarios of aircraft near the ground passing through the light path at the airport.
